# A robust salt-tolerant superoleophobic alginate/graphene oxide aerogel for efficient oil/water separation in marine environments

**DOI:** 10.1038/srep46379

**Published:** 2017-04-11

**Authors:** Yuqi Li, Hui Zhang, Mizi Fan, Peitao Zheng, Jiandong Zhuang, Lihui Chen

**Affiliations:** 1 College of Materials Engineering, Fujian Agriculture and Forestry University, Fuzhou 350002, China; 2 Nanocellulose and Biocomposites Research Centre, College of Engineering, Design and Physical Sciences, Brunel University, UB8 3PH, UK

## Abstract

Marine pollution caused by frequent oil spill accidents has brought about tremendous damages to marine ecological environment. Therefore, the facile large-scale preparation of three-dimensional (3D) porous functional materials with special wettability is in urgent demand. In this study, we report a low-cost and salt-tolerant superoleophobic aerogel for efficient oil/seawater separation. The aerogel is prepared through incorporating graphene oxide (GO) into alginate (ALG) matrix by using a facile combined freeze-drying and ionic cross-linking method. The 3D structure interconnected by ALG and GO ensures the high mechanical strength and good flexibility of the developed aerogel. The rough microstructure combined with the hydrophilicity of the aerogel ensures its excellent underwater superoleophobic and antifouling properties. High-content polysaccharides contained in the aerogel guarantees its excellent salt-tolerant property. More impressively, the developed aerogel can retain its underwater superoleophobicity even after 30 days of immersion in seawater, indicating its good stability in marine environments. Furthermore, the aerogel could separate various oil/water mixtures with high separation efficiency (>99%) and good reusability (at least 40 cycles). The facile fabrication process combined with the excellent separation performance makes it promising for practical applications in marine environments.

In the past decades, the marine pollution caused by frequent oil spill accidents has brought about tremendous damages to marine ecological environment^1^,^2^,^3^. How to efficiently and effectively realize oil/water separation in marine environments has become a subject of global concern. Since oil/water separation is an interfacial issue, applying functional materials with special wetting properties is considered an effective and facile strategy^4^,^5^,^6^,^7^,^8^. Up until now, most in-air superoleophobic materials are based on the modification of fluorination reagents^9^,^10^,^11^. However, fluoride-modified materials will lose their superoleophobicity in seawater. Encouragingly, the discovery of unique underwater oil-repellent property of fish scale opened a new window for fabricating underwater superoleophobic materials such as hydrogels coatings^12^,^13^,^14^,^15^ and inorganic metal oxide coatings^16^,^17^,^18^,^19^. However, the biggest challenge of using these materials is their poor environmental adaptability. They may fall off from their substrates or suffer from swelling deformation and result in a gradual loss of superoleophobicity after a long-term seawater immersion. Furthermore, current methods are complicated, expensive, and difficult to realize a large-scale production. Therefore, it is imperative to develop function materials that are cost-effective, salt-tolerant and scalable to achieve the oil/water separation in marine environments.

Aerogel, a kind of highly porous solid material with three-dimensional (3D) network structure and low density, has been considered to be a promising candidate for achieving an efficient oil/water separation in a gravity-driven process with high-flux^20^,^21^,^22^. Alginate (ALG) is a linear, unbranched polysaccharide consisting of (1, 4)-linked α-L-guluronic acid units (G-blocks) and β-D-mannuronic acid units (M-blocks)^23^. It is naturally present in the cell walls of brown seaweeds and commercially available as a sodium salt^24^. Recent studies have indicated that this low-cost natural biomaterial possesses excellent salt-tolerant and underwater superoleophobic properties^25^. Thus, alginate aerogel, combining the advantages of both, has great potentials to achieve oil/water separation in marine environments. However, there are still two obstacles to overcome, namely (i) the low mechanical strength of pure alginate aerogel and (ii) complicated ionic cross-linking process. To overcome the first obstacle, an effective approach is the incorporation of reinforcing fillers into the alginate matrix and here graphene oxide (GO) is chosen as reinforcing filler owing to its excellent mechanical property and remarkable flexibility^26^,^27^,^28^, which can effectively resist the load transferred from the alginate matrix. It is well known that directly introducing divalent ions into alginate solution is more likely to produce anisotropic gel particles instead of a homogeneous gel, which attributes to the rapid cross-linking reaction between the carboxyl groups of the alginate and divalent ions. To tackle this problem, the CaCO_3_-GDL (Gluconolactone) system developed by Kuo *et al*. was usually introduced to reduce the release rate of calcium ions^29^,^30^,^31^,^32^. Although this system is available to produce homogenous alginate gel, it is more complex in practical operation. Therefore, in response to the second obstacle, this study presents a more facile method by which the ALG/GO mixture was firstly freeze-dried to obtain uncross-linked aerogel scaffold and then immersed in CaCl_2_ aqueous solution to achieve the rapid ionic cross-linking reaction. In other words, the method takes full advantage of the rapid reaction between alginate and calcium ions to quickly harden the porous ALG/GO scaffold.

Herein, a low-cost and robust alginate (ALG)/graphene oxide (GO) hybrid aerogel was fabricated via a facile combined freeze-drying and ionic cross-linking method (Fig. 1). The developed ALG/GO aerogel without any further chemical modification was, to our knowledge, used for oil/seawater separation for the first time. The characteristics of the ALG/GO aerogel, including wetting behaviours in air and seawater, salt tolerance, mechanical performance, separation efficiency and recyclability for oil/seawater mixtures were investigated in detail. As expected, the developed hybrid aerogel exhibited high separation efficiency and good recyclability for oil/seawater mixtures, making it promising for practical applications in marine environments.

## Results and Discussion

**Figure 2:**
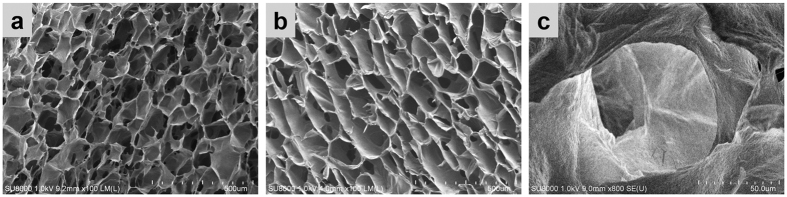
FE-SEM images of cross sections (**a**), vertical sections (**b**), and high magnification (**c**) of the ALG/GO aerogel.

The microstructural morphology of the ALG/GO aerogel was studied using a scanning electron microscopy (SEM). As illustrated in Fig. 2, the aerogel exhibits a fine pore structure, similar to a sponge or crumb of bread. There is no obvious difference between structures on the cross sections (Fig. 2a) and vertical sections (Fig. 2b). Quite evidently, the aerogel is highly porous and has well-connected 3D network structures. The pore size of the aerogel is randomly distributed in the range of 50–150 μm. The ideal pore size and network-distribution of the as-prepared aerogel allow oil/water separation to be achieved in a gravity-driven process with high-flux. The highly-magnified SEM image (Fig. 2c) shows the micropores in aerogel are irregular in shape and have a rough surface. The surface roughness combined with its high affinity to water is in fact essential for achieving underwater superoleophobic property of the aerogel.

**Figure 3:**
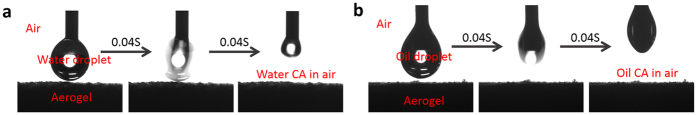
Water wettability (**a**) and oil wettability (**b**) of ALG/GO aerogel in air.

To investigate the wetting behaviour of ALG/GO aerogel towards water and oil in air, a high-resolution camera was used to record the spreading of water and oil droplets. When a water droplet (5 μL) contacts the aerogel surface in air, it spreads out and permeats into the aerogel quickly, resulting in a contact angle of approximately 0° (Fig. 3a). A similar phenomenon is also observed when an oil droplet (5 μL) contacts the aerogel surface (Fig. 3b). The superamphiphilic property of the aerogel in air may be attributed to the cooperative effect of its surface rough microstructure and large amounts of hydroxyl groups on the aerogel surface.

**Figure 4:**
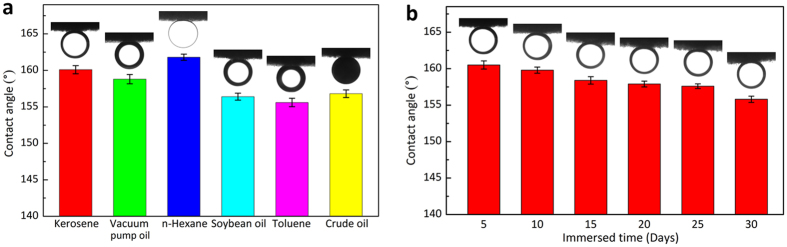
(**a**) the contact angles of various oil on ALG/GO aerogel under seawater; (**b**) the underwater contact angles of kerosene on ALG/GO aerogel during 30 days of immersion in seawater.

The underwater superoleophobicity of the ALG/GO aerogel was investigated by immersing the aerogel into seawater. In the three-phase system of oil/water/solid, the water trapped in the micro-roughness structures of ALG/GO aerogel gives rise to a strong repulsive force to oil, resulting in a large contact angle. Therefore, without any chemical modification, the as-prepared ALG/GO aerogel possesses underwater superoleophobic property. The measuring process of underwater oil contact angle was recorded in Supplementary Movie S1. As shown in the movie, the underwater OCA of ALG/GO aerogel is as large as 160.1°, indicating the aerogel has excellent underwater superoleophobic property. Meanwhile, the oil droplet (kerosene) would shake and quickly slide off the aerogel surface when it was disturbed slightly. The low affinity of the water-wetted aerogel for oil can effectively prevent ALG/GO aerogel to be polluted or blocked up by oil during oil/water separation process. In addition, the aerogel also shows extraordinary underwater superoleophobic property for other oils and organic solvents (Fig. 4a). The oil contact angles for crude oil, kerosene, n-hexane, toluene and soybean oil are all larger than 155° in seawater.

To evaluate the environmental stability, the contact angle of aerogel was measured after being exposed to seawater for a prolonged period of time (Fig. 4b). It is apparent that the aerogel retains its underwater superoleophobic property (OCA > 150°) after soaking for 30 days, indicating an excellent durability in high ionic strength environments, which ascribes to its main content of polysaccharides and robust 3D interconnected network structure.

From the viewpoint of practical applications, the mechanical strength and flexibility of the ALG/GO aerogel also play important role in the separation of oil/seawater mixture. As shown in Supplementary Movie S2, the aerogel can be crumpled into a ball and recovered immediately to its original shape without any damage, which mainly due to its mechanical stability of 3D cross-linked structures.

To take full advantage of the above properties, the ALG/GO aerogel was used to perform gravity-driven oil/seawater separation. The experiment was carried out as illustrated in Supplementary Movie S3. The ALG/GO aerogel was fixed between two glass tubes by stainless steel spring clamps and then a mixture of oil and seawater (1:1, v/v) was poured into the upper tube. The seawater quickly penetrated through the pre-wetted aerogel by gravity, while the oil was selectively blocked. There was no visible oil observed in the filtered water, indicating an efficient separation of the oil/seawater mixture.

**Figure 5:**
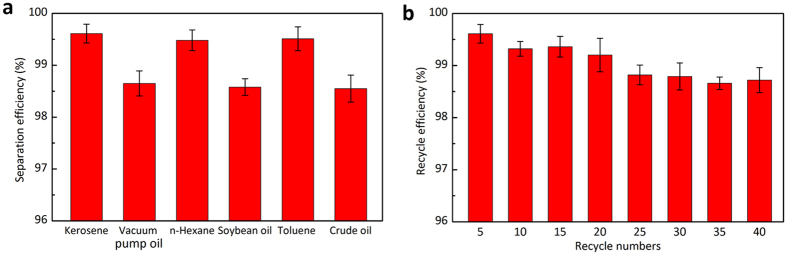
(**a**) the separation efficiency of oil/seawater mixtures; (**b**) the separation efficiency versus the recycle numbers by taking the kerosene/seawater mixture as an example.

The separation efficiency *η* of the aerogel was also confirmed by the following eqn (1):


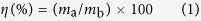


where, *m*_b_ and *m*_a_ represent the mass of the oil before and after filtration, respectively. The separation efficiency was calculated to be above 99.6% for the kerosene-seawater mixture and above 98.5% for other oils and organic solvents (Fig. 5a). The efficiency of the separation may be attributed to the high porosity and underwater superoleophobicity of the aerogel. Moreover, the reusability of ALG/GO aerogel has been studied. It has been found that the separation efficiency for the kerosene-seawater mixture remains above 98.5% after the aerogel has been reused for 40 separation cycles (Fig. 5b), confirming the excellent reusability of the as-prepared aerogel.

To further study the separation ability of the ALG/GO aerogel, Oil intrusion pressure and water flux were also investigated. Oil intrusion pressure, also called oil breakthrough, essentially caused by repulsive force between the water-wetted aerogel and oil in the oil/water/solid three-phase system, macroscopically reflected the maximum height (*h*_max_) of oil that the as-prepared aerogel can support.

**Figure 6:**
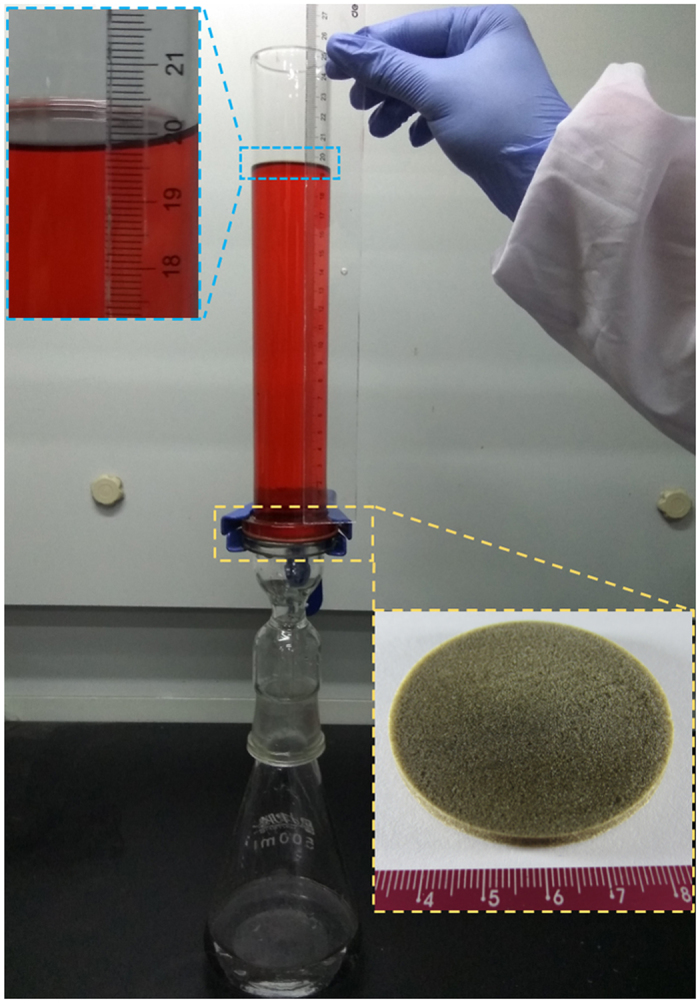
The intrusion pressure of oil taking kerosene as an example.

The oil intrusion pressure (*P*_o_) was calculated using eqn (2):


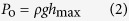


where, *ρ* is the density of oil, *g* is the acceleration of gravity, and *h*_max_ represents the maximum height of oil that the aerogel can support^33^. As displayed in Fig. 6, the value of *h*_max_ can reach 19.8 cm for kerosene and the oil intrusion pressure is 1.55 kPa. Under this pressure, the aerogel can achieve the separation of kerosene/seawater mixture.

The water flux (*F*), defined as the volume of water that passes through the aerogel per unit time, was calculated according to eqn (3):


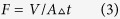


where, *V* is the volume of seawater that permeates through the aerogel, *A* is the effective filtration area of the aerogel and ▵t represents the time required for the permeation. Here, the water flux calculated from eqn (3) was as high as 3.8 L m^−2^ s^−1^.

To illustrate the water/oil separation mechanism of the ALG/GO aerogel, the equation to calculate theoretical intrusion pressure was introduced^34^,^35^,^36^.


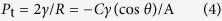


where *γ* is the surface tension, *θ* is the liquid contact angle on the material, *R* is the radius of the meniscus, *C* is the circumference of the pore in the material, and A is the cross-sectional area of the pore. According to eqn (4), the intrusion pressure *P*_t_ is >0 (negative capillary effect) when *θ > *90°. Under this condition, the material can sustain a certain liquid pressure. In other words, liquid cannot permeate the aerogel spontaneously without an external pressure. In contrast, the intrusion pressure *P*_t_ is <0 (capillary effect) when *θ* < 90°. In this case, materials cannot support any pressure and liquid would permeate the aerogel spontaneously by self-gravity. From the discussion in the previous sections, it is apparent that the superamphiphilicity (*θ* is nearly 0°) of the aerogel allows liquid (water or oil) to permeate through the aerogel quickly by self-gravity. When the aerogel was immersed in water, water would trap in the rough microstructure of the aerogel that then formed an oil/water/solid three-phase system in the presence of oil. The repulsive force between the trapped water and oil resulted in a significant reduction in the contact area between the oil and surface of the aerogel, leading to *θ* > 150° (underwater superoleophobicity) and *P*_t_ > 0, hence oil/water mixtures could be selectively separated efficiently using the developed underwater superoleophobic ALG/GO aerogel.

In conclusion, a robust salt-tolerant superoleophobic aerogel has been developed through incorporating graphene oxide (GO) into alginate (ALG) matrix by combining freeze-drying and ionic cross-linking process. The 3D interconnected network structure of the ALG/GO aerogel was obtained, which ensured its high mechanical strength and good flexibility. The developed aerogel showed excellent underwater superoleophobicity, salt-tolerance and antifouling properties. The aerogel can be adapted to perform different types of oil/seawater separation and demonstrated both highly efficient and reusable in high-salinity environments. We believe that this kind of aerogel is a promising candidate material for oil/water separation in marine environments.

## Methods

### Materials

Graphene oxide (GO) suspensions (1 wt%) were obtained from Shanghai Ashine Technology Development Co., Ltd. Sodium alginate was purchased from Shanghai Macklin Biochemical Co., Ltd. Calcium chloride (CaCl_2_) was purchased from Xilong Chemical Co., Ltd. Crude oil was provided by Befar Group Co., Ltd. Toluene and n-hexane were purchased from Shanghai Aladdin Bio-Chem Technology Co., Ltd. Kerosene was obtained from SINOPEC Fujian Refining & Chemical Co., Ltd. Vacuum pump oil was purchased from ExxonMobil (China) Investment Co., Ltd. Soybean oil was obtained from Shandong Luhua Group Co., Ltd. Sea salts were purchased from Sigma Aldrich, which is an artificial salt mixture closely resembling the composition of the dissolved salts of ocean water. The seawater was prepared by dissolving 17.2 g sea salts in 500 mL of deionized water.

### Preparation of alginate (ALG)/graphene oxide (GO) aerogel

The ALG/GO aerogel was fabricated through incorporating graphene oxide (GO) into alginate (ALG) matrix by combining freeze-drying and ionic cross-linking process. Firstly, the GO suspension (1 wt%) was ultrasonic-dispersed for 15 min to obtain a homogeneous dispersion. Meanwhile, sodium alginate was dissolved in deionized water and constantly stirred for 2 h to form a transparent solution (2 wt%). Then, GO suspensions were added into the above sodium alginate solution in a volume ratio of 1: 12 (GO suspensions: sodium alginate solution), with continuous magnetic stirring, until a homogeneous dispersion was obtained. The sodium alginate/graphene oxide mixture was poured into a Teflon Petri dish, frozen at −18 °C, and lyophilized at 600 mbar (absolute pressure) for 40 h in a laboratory-scale freeze dryer (LyoBeta 3PS, Telstar, Spain) to produce the aerogel. After that, the aerogel was immerged in 5 wt% CaCl_2_ solution for 10 h to achieve the calcium ion induced cross-linking process. After cross-linked by calcium ions, the aerogel was washed with deionized water several times to remove the unbound calcium ions and freeze-dried again to obtain the cross-linked ALG/GO aerogel.

### Oil/seawater separation experiment

Six types of oils and organic solvents, including kerosene, toluene, n-hexane, vacuum pump oil, soybean oil and crude oil were dyed red and respectively mixed with seawater to prepare different kinds of oil/water mixtures. The ALG/GO aerogel with 40 mm diameter and 6 mm thickness was carefully fixed between two glass tubes by stainless steel spring clamps. Then, a mixture of oil and seawater (1:1, v/v) was poured into the upper tube. Finally, the separation process was achieved by the driving force of gravity.

### Characterization methods

The surface morphologies of the aerogel were assessed using field emission scanning electron microscopy (FE-SEM, Hitachi SU8000). The water and oil contact angles were measured using a drop shape analysis system (Kruss DSA30) by injecting 5 μL of water or oil droplets onto the ALG/GO aerogel surfaces. Five different locations of the surface were examined to obtain the average value.

## Additional Information

**How to cite this article:** Li, Y. *et al*. A robust salt-tolerant superoleophobic alginate/graphene oxide aerogel for efficient oil/water separation in marine environments. *Sci. Rep.* **7**, 46379; doi: 10.1038/srep46379 (2017).

**Publisher's note:** Springer Nature remains neutral with regard to jurisdictional claims in published maps and institutional affiliations.

## Supplementary Material

Supplementary Movie S1

Supplementary Movie S2

Supplementary Movie S3

Supplementary Movie Captions

## Figures and Tables

**Figure 1:**
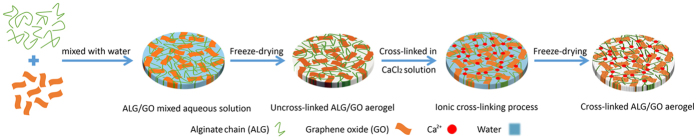
The preparation of cross-linked alginate (ALG)/graphene oxide (GO) aerogel.
