# Supplementary material for: A robust salt-tolerant superoleophobic alginate/graphene oxide aerogel for efficient oil/water separation in marine environments
Source: Sci Rep. 2017 Apr 11;7:46379. doi: 10.1038/srep46379 (PMC5387746; doi:10.1038/srep46379)
Supplement: Supplementary Movie Captions [file srep46379-s4.doc]

**A robust salt-tolerant superoleophobic alginate/ graphene oxide aerogel for efficient oil/water separation in marine environments**

**Yuqi Li^1,+^, Hui Zhang^1,+^, Mizi Fan^1,2,*^, Peitao Zheng^1^, Jiandong Zhuang^1^ and Lihui Chen^1^**

^1^College of Materials Engineering, Fujian Agriculture and Forestry University, Fuzhou 350002, China.

^2^Nanocellulose and Biocomposites Research Centre, College of Engineering, Design and Physical Sciences, Brunel University, UB8 3PH, UK.

^*^E-mail: mizi.fan@chemteam.cn; mizi.fan@brunel.ac.uk

^+^These authors contributed equally to this work.

**Supplementary Movie Captions**

**Movie S1.** The measuring process of underwater oil contact angles for kerosene.

**Movie S2**. The illustration of mechanical strength and flexibility of the ALG/GO aerogel.

**Movie S3.** The separation process of oil (kerosene)/seawater mixture based on the ALG/GO aerogel.
